# Ultra-high-throughput Production of III-V/Si Wafer for Electronic and Photonic Applications

**DOI:** 10.1038/srep20610

**Published:** 2016-02-11

**Authors:** Dae-Myeong Geum, Min-Su Park, Ju Young Lim, Hyun-Duk Yang, Jin Dong Song, Chang Zoo Kim, Euijoon Yoon, SangHyeon Kim, Won Jun Choi

**Affiliations:** 1 Korea Institute of Science and Technology (KIST), Hwarangno 14-gil 5, Seongbuk-gu, Seoul, 136-791, Korea; 2 Department of Materials Science and Engineering, Seoul National University, Gwanak-gu, Seoul, 151–742, Korea; 3 Korea Photonics Technology Institute (KOPTI), Cheomdan venture-ro 108-gil 9, Buk-gu, Gwanju-si, 500-799, Korea; 4 Korea Advanced Nanofab Center (KANC), Gwanggyo-ro 109, Yeongtong-gu, Suwon-si, Gyeonggi-do, 443-270, Korea

## Abstract

Si-based integrated circuits have been intensively developed over the past several decades through ultimate device scaling. However, the Si technology has reached the physical limitations of the scaling. These limitations have fuelled the search for alternative active materials (for transistors) and the introduction of optical interconnects (called “Si photonics”). A series of attempts to circumvent the Si technology limits are based on the use of III-V compound semiconductor due to their superior benefits, such as high electron mobility and direct bandgap. To use their physical properties on a Si platform, the formation of high-quality III-V films on the Si (III-V/Si) is the basic technology ; however, implementing this technology using a high-throughput process is not easy. Here, we report new concepts for an ultra-high-throughput heterogeneous integration of high-quality III-V films on the Si using the wafer bonding and epitaxial lift off (ELO) technique. We describe the ultra-fast ELO and also the re-use of the III-V donor wafer after III-V/Si formation. These approaches provide an ultra-high-throughput fabrication of III-V/Si substrates with a high-quality film, which leads to a dramatic cost reduction. As proof-of-concept devices, this paper demonstrates GaAs-based high electron mobility transistors (HEMTs), solar cells, and hetero-junction phototransistors on Si substrates.

Historically, III-V compound semiconductors have been explored as active materials for high-speed electronic devices^1^,^2^, high-efficiency photovoltaic devices^3^,^4^, and many types of opto-electronic devices^5^,^6^. The widespread use of III-V semiconductors is due to the inherent advantages of direct bandgap and high electron mobility. However, in spite of their superior properties, the main bottlenecks to mass-production of III-V devices are the high wafer cost and the limited wafer size. To circumvent these problems, the concept to integrate III-V films on Si substrates appears to be quite promising. Similarly, to extend Si technology, a method of high-quality III-V/Si formation is a key issue to leverage the benefits of III-V materials and the Si platform. Early attempts to form the III-V/Si substrate have used various epitaxial growth techniques, such as global epitaxial growth^7^,^8^,^9^, aspect ratio trapping^10^,^11^, and lateral overgrowth^12^,^13^, etc. However, all these growth techniques have suffered from a poor epitaxial layer quality (high defect density) and an integration complexity with conventional Si devices. Recent few developments show a relatively high film quality with almost no anti-phase boundary defects or dislocation defects^14^,^15^.

Recently, high-quality III-V/Si formation method based on wafer bonding have been rapidly developed by many research groups, with the successful production of high-quality III-V films on Si substrates^5^,^16^,^17^,^18^,^19^,^20^. We also reported GaAs solar cells and hetero-junction phototransistors (HPTs) on Si via transfer of a high-quality film through the wafer bonding process^21^,^22^,^23^. However, in many cases, these demonstrations are only feasibility studies, because they involve etching of an entire donor substrate after the formation of the III-V/Si substrate. Such etching is extremely costly and cannot be directly used in a mass-production process. Several trials to re-use the donor wafer via hydrogen-induced wafer splitting have been conducted^17^,^24^; however, this approach leaves many defects in the III-V film, which is harmful to device reliability.

Another approach to wafer re-use is the use of epitaxial lift off (ELO) techniques. ELO was developed in the 1980s and 1990s^25^,^26^ after Konagai *et al.* first proposed the method^25^, in which the device film and donor wafer are split by the selective etching of the AlGaAs layer located between the device film and the donor wafer. However, ELO was not popularly used at the time due to very long process time of up to several days. Recently, a reduction of the processing time for the ELO process has been developed by introducing a flexible carrier substrate and a different sacrificial layer; however, the handling of the flexible carrier increases the process complexity and still requires a long processing time of several hours^27^,^28^. Therefore, for the practical use of the ELO technique, it is important to reduce the processing time and produce a high-quality film at the same time.

In this work, we describe an ultra-fast ELO process that involves a pre-patterning step before the wafer bonding and the use of etching acceleration solutions. Furthermore, we demonstrate that the III-V donor wafer can be re-used after the wafer bonding and ELO process at least once. These approaches enable a high-throughput fabrication of GaAs/Si substrate with a high-quality film of GaAs, which lead to a dramatic cost reduction, as estimated in Supplementary Information.

Figure 1 shows a schematic fabrication process of the GaAs/Si substrate using the proposed wafer bonding and ELO approach (See method for the details). We inserted the etch stop layer of InGaP at the bottom and the top of the Al_0.85_Ga_0.15_As sacrificial layer to protect the active layer and the GaAs donor wafer during the ELO process. The main cause of the long processing time for the ELO was known to be the formation of H_2_ bubbles and the etching residues generated during the ELO process^28^,^29^,^30^. To encourage H_2_ bubble release and increase the accessible etching areas, a pre-patterning step that breaks the III-V layer into smaller pieces was added between the growth of III-V layer and the wafer bonding. Actually, the use of the patterned channel was reported for the outgassing in III-V/Si hybrid structure, which enhanced thermal stability due to efficient gas release through the channels^31^,^32^,^33^,^34^. In addition, we inserted etching acceleration solutions into HF, which is typically used for the etching of the Al_0.85_Ga_0.15_As sacrificial layer in the ELO process. As we explained afterwards, etching acceleration solutions were isopropanol (IPA) and acetone (Ace), which accelerate the etching of Al_0.85_Ga_0.15_As sacrificial layer by making the H_2_ gas release smoothly and reducing the etch residues. Here, one possible concern is the surface contamination during the patterning and etching. Therefore, for the high yield bonding, the careful surface preparation before the wafer bonding is needed. In our experiment, overall transfer yield was higher than 95%. Next, the wafer bonding was conducted at room temperature, followed by the device fabrication (See method and Supplementary Information). To reduce the unfavorable effect due to the difference of the thermal expansion, a processing temperature including wafer bonding and device fabrication should be minimized (See Fig. S2, Supplementary Information). After this series of steps of the process, the donor wafer was re-used for another epitaxial growth using a flat and fresh surface formed by a highly selective etching of an etch stop layer (InGaP) using HCl:H_3_PO_4_ solutions.

Figure 2a shows the infrared (IR) photograph of GaAs on insulator (-OI) on a Si wafer after the wafer bonding with a pre-patterning step. Here, Y_2_O_3_ was used as the bonding material^21^. Clear bonding behaviors were observed in the IR image over the 2-inch full wafer scale. The top-side view of the scanning electron microscopy (SEM) image of the GaAs/Si wafer after the ELO is shown in Fig. 2b. The image confirmed that the GaAs pattern arrays were bonded onto the Si wafer with the same shape as defined in the pre-patterning step. A cross-sectional transmission electron microscopy (TEM) image in Fig. 2c reveals that the GaAs high electron mobility transistors (HEMTs) was bonded onto the Si with good uniformity and without any voids at the bonding interface. Here, the active layer for GaAs HEMT was considered; however, this approach is not limited to HEMT devices and can be expanded to any possible set of active layers. Energy dispersive x-ray (EDAX) spectroscopy in Fig. 2d shows the abrupt interfaces between GaAs, Y_2_O_3_, and Si. Fig. 2e,f depict the transmission electron diffraction patterns in the bonded GaAs and the Si, respectively. The patterns show that both GaAs and Si are single crystals in structure, and crystalline structures of the bonded III-V layer were maintained during the wafer bonding and ELO process. Also, from the lattice spacing (d), the lattice constant of GaAs and Si was well-matched to the crystallographic data of GaAs and Si, respectively. These results strongly indicate that the wafer bonding and ELO techniques produced a high-quality III-V layer on the Si substrate.

To investigate the effect of the pre-patterning step and the etching acceleration solutions, we compared the ELO process for various etching conditions. Figure 3a illustrates the ELO time for different etching conditions. Here, pieces of the bonded substrate of GaAs/Al_0.85_Ga_0.15_As/GaAs/Y_2_O_3_/Si with a size of 1.5 × 1.5 cm^2^ were used for these experiments. For the pre-patterning, a mesa size of 670 × 620 μm^2^ and spacing of 230 μm were used as standard dimensions. Without a pre-patterning step, the ELO process takes 30 hours in HF:DIW (1:5) solutions, which directly shows the difficulty of the use of a conventional ELO process. With a pre-patterning step, the ELO time was significantly reduced to 6 hours in the same solutions. This reduction is attributed to the increase of an exposed surface area of an Al_0.85_Ga_0.15_As layer due to the pre-patterning, which enabled the efficient gas bubbles release^31^,^32^,^33^,^34^, resulting in the reduction of the ELO time. Here, it is important issue in our technology to investigate the dependence of the size of the transferred structure. Significant ELO time reduction was obtained with a decrease of the mesa size due to an increased exposed surface area, whereas there was almost no spacing dependence of the ELO time, indicating that more dense packing will be possible by choosing appropriate mesa and spacing size (Fig. S4). To further reduce the ELO time, we changed the HF concentration and added etching acceleration solutions of isopropanol (IPA), acetone (Ace). Here, organic solutions, such as IPA and Ace, are known to produce hydrophilic surfaces^35^, which can prevent H_2_ bubbles from becoming large. This process promotes the rapid release of H_2_ bubbles from the samples. With increasing HF concentration and the addition of etching acceleration solutions, the ELO time was significantly reduced down to approximately 20 min. Comparing to that of other study with a channel release of 110 min, the ELO time achieved in this study was quite short^18^. These results strongly suggest that a pre-patterning step and the addition of etching acceleration solutions facilitate high-throughput III-V/Si wafer fabrication. To understand the physical origin of the effect of the etching acceleration solutions, we measured the residue thickness via an ellipsometry measurement after the immersion of the samples in various solutions. The measurement was conducted after 6 hours of immersion in each solution. In many studies, the residue, e.g., As during the ELO process was known to be the reaction barrier for a succeeding lateral etching, because it makes solution flowing difficult^29^,^30^,^31^,^32^,^33^,^34^,^35^. Consistently, with the results of the ELO time, the residue thickness decreases in the order of HF:DIW > HF:IPA > HF:Ace. As a result, simply, we would be able to regard the enhancement factor of the pre-patterning and the insertion of the solution as 5×(30 hours/6 hours) and 18× (6 hours/20 min), respectively. Next, the wafer size dependence was investigated for our ELO process. Although slight run-to-run variations were observed in our experiments, the ELO time was almost constant with increasing the wafer size, whereas a typical ELO process requires an exponential increase in the processing time with increasing the wafer size. The pre-patterned mesas promote the flowing of etching solutions during the chemical reactions. Further ELO time reduction will be possible by making mesa pattern with longer perimeter and also using the solution circulation and/or vapor phase etching scheme, which is typically used in micro electro mechanical systems (MEMS) technology to promote an etchant flowing^36^.

Figure 3d shows the surface morphology of the GaAs surface at each of the process steps characterized using atomic force microscopy (AFM). A bare epi-ready GaAs wafer shows a flat surface with a root mean square roughness (R_rms_) of 0.02 nm. After the ELO process, the R_rms_ of the surface was increased up to 0.5 nm and etching residues with dot shapes were observed over the entire wafer. After the cleaning using HCl and the etching of the etch stop layer, the AFM images illustrate the excellent surface morphology that is comparable to that of the epi-ready surface. The AFM image of the surface after re-growing the GaAs layer confirmed a high process stability with a flat and smooth surface. To investigate the layer quality and the possible strain in the transferred film, we collected Raman spectra, as shown in Fig. 3e. A high-quality film was maintained during the wafer bonding and ELO process. The peak of the Si in the Raman spectra was also observed from the GaAs/Si substrate, confirming the fabricated structure. A negligible change of the peak position was observed, indicating the transferred film has no strain. The Raman spectra of the donor wafer after the ELO also indicate a high-quality film. Even after the re-growth on the donor wafer formerly used in the ELO process, the Raman spectra was still sharp and no strain was observed in the re-grown layer. However, the Raman spectra after re-growth seems to be broader than that of the initially grown GaAs layer, indicating the crystal quality of re-grown GaAs layer seems to be not perfect. It is possibly because that the surface treatment including wet and thermal treatment before the regrowth has not been optimized, whereas surface chemistry strongly impacts the crystal quality of III-V films^27^,^37^.

To characterize the performance of the electron devices on the III-V/Si using our wafer bonding and ELO process, we fabricated conceptual devices of the GaAs-OI HEMTs on Si (see the details in Figs S1 and S2). After growing the inverted AlGaAs/GaAs HEMT structure with an Al_0.85_Ga_0.15_As sacrificial layer between a device layer and the GaAs donor wafer (see the details in Fig. S1), Y_2_O_3_ was deposited as a bonding material, and the pre-patterning process was then performed. Due to the insertion of the pre-patterning, mesa isolation before the device fabrication can be skipped afterward. Here, the surface of Y_2_O_3_ was very flat, and sufficiently smooth for the wafer bonding process (see the details in Fig. S3). Next, the wafer bonding and ELO processes were conducted in HF:Ace solutions. After splitting the GaAs-OI HEMT on Si and the GaAs donor wafer, GaAs-OI HEMT was completed via the following step: Pd/Ge/Au ohmic metallization, gate recess, and Ti/Pt/Au gate deposition (see the details in Fig. S4). Device characterization was performed at room temperature in the dark.

Figure 4a shows the typical drain current (*I*_D_)-gate voltage (*V*_G_) characteristics of the GaAs-OI HEMT with a gate length (*L*_G_) of 2 μm. Clear transfer curves were obtained with a steep subthreshold slope (*S*.*S*. = d*V*_G_/d[log(*I*_D_)]) of 83 mV per decade and high on/off ratio (*I*_on_/*I*_off_) of 10^7^. To the best of our knowledge, the achieved *S*.*S*. and *I*_on_/*I*_off_ are record values among the reported III-V-OI transistors^17^,^18^,^19^,^20^,^21^. In addition, *I*_off_ was found to be a very low value of <1 pA/μm, which is lower than that required for an ultra-low power (ULP) transistor (10 pA/μm) by the International technology roadmap for semiconductors (ITRS). The clear current saturation was also observed in the *I*_D_-drain voltage (*V*_D_) characteristics (Fig. 4b). From the structural specialty of having a back-gate structure through the Y_2_O_3_ insulating film, the electrical properties of the fabricated GaAs-OI HEMT can be modulated by the biasing of the back gate (*V*_B_) of the Si substrate. Since GaAs channel layer and underlying Si substrate are electrically isolated, a channel potential can be modulated via *V*_B_ biasing without a leakage current. Fig. 4c shows the *I*_D_-*V*_G_ curves with changing *V*_B_ from 0.5 to −0.5 V. The *I*_D_-*V*_G_ curves were intentionally shifted with changing *V*_B_, indicating that the device characteristics can be controlled after the device fabrication, which provides additional functionality for a circuit design.

Even though the re-grown GaAs layer on the donor wafer after the ELO process was evaluated by AFM and Raman spectra, these results do not guarantee the re-use of the donor wafer because these measurements do not fully reflect the epitaxial quality. Here, the best way to show the wafer-reusability is to fabricate and compare each types of device. However, for the simple examination, Hall mobility is the one of the best figure-of-merit to evaluate the crystal quality of grown epitaxial film, which is typically used to check/confirm the status of the growth chamber. Therefore, we have investigated the electrical properties of re-grown HEMTs. Figure 4d shows the Hall mobility and charge carrier density (*N*_s_) of fresh and re-grown GaAs HEMTs at 80 K and 300 K. The mobility and *N*_s_ behaviors were almost the same between the two sets of devices, which indicates the high quality of the re-grown GaAs HEMT layer, whereas the Raman spectra was slightly broader. These results strongly suggest that the epi-ready surface of the GaAs donor wafer can be recovered after the ELO process, thereby enabling the donor wafer to be re-used at least once. Further development will be expected via the optimization of the surface treatment condition before the re-growth with a quality evaluation by the Raman spectra, Hall mobility, and each device performance.

For photonic devices, we demonstrated GaAs single-junction solar cells and InGaP/GaAs HPTs on Si substrate. Similar to the HEMT fabrication process, an inverted epitaxial structure consists of a GaAs base and emitter and an InGaP back surface field, window layer, which was grown with an Al_0.85_Ga_0.15_As sacrificial layer, followed by Pt/Au deposition (see the details in Fig. S1). Here, we used Pt/Au as the bonding material as demonstrated in our previous study^23^. Therefore, GaAs and Si substrate were electrically conductive unlike the case of using Y_2_O_3_ bonding material. Subsequently, the pre-patterning was performed, followed by the wafer bonding and ELO process. Next,the fabrication of the GaAs solar cells on Si was completed by the top grid (Pt/Ti/Pt/Au) formation and etching of the GaAs top contact layer (see the details in Fig. S5). Here, we fabricated micrometer-scale solar cells, as shown in the inset of Fig. 5a, which is suitable for the developed ELO process in this study (see more images in Fig. S6). One cell was defined as a pre-patterned mesa before the wafer bonding process, resulting in process simplification by using the pre-patterned mesa for the device isolation. One concern of the use of our ELO method for solar cell fabrication is the limitation of the size of devices for the large-scale solar panel. After forming solar cell arrays on the Si, an electrical connection of each cell arrays through the metal contact on the pre-patterned mesa spacing will be efficient way for the scale-up of the panel size^38^,^39^. Placing the metal contacts on the pre-patterned mesa spacing, additional space penalty can be released.

Figure 5a shows the current density (*J*) – *V* curves of the GaAs solar cell on Si under air mass (AM) 1.5 G, 1-sun measurement conditions. As a control device, the data of a GaAs solar cell grown on GaAs is also shown. Even considering approximately 30% reflection of the incident light due to the absence of an anti-reflection coating, the energy conversion efficiency (*η*) and the open-circuit voltage (*V*_oc_) of the solar cell on Si was as high as 14.05% and 0.89 V, respectively. The performance of the solar cell on Si was almost same as that of the control device with an even higher short-circuit current (*J*_sc_). The external quantum efficiency (EQE) of both devices in Fig. 5b shows no substantial difference, whereas *J*_sc_ value is different between the two. We believe that light reflection from the back side enhances photo recycling of the light once reached to the back-side, leads to higher *J*_sc_ in the solar cell on Si. However, since the intensity of an incident light to the sample was very small in EQE measurement, the data could not catch the light response reflected from back side metal. These results strongly suggest that the developed wafer bonding and ultra-fast ELO techniques provide a high-quality III-V film on Si substrate without material degradation during these processes.

GaAs HPTs on Si were also fabricated using similar procedures. After growing an epitaxial layer comprising a GaAs collector, a GaAs base, and an InGaP emitter, the III-V and Si wafers were bonded to each other using a Pt/Au bonding layer, followed by the ELO (see the details in Fig. S1). Next, the fabrication of HPTs was completed with the steps of the ohmic metallization (Ni/Au/Ge/Ni/Au) for the collector and the mesa etching (see the details in Fig. S7). Figure 5d shows the collector dark current (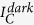) and the collector photocurrent (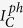) characteristics of a fabricated InGaP/GaAs HPT on Si as a function of the bias voltage across the collector and the emitter (*V*_CE_). 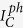 was measured with an incident optical power (*P*_in_) of 1.2 μW at a wavelength of 635 nm. The data from the HPTs fabricated by both the developed ultra-fast ELO and the conventional ELO without the pre-patterning and accelerant addition are shown. For both devices, 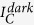 was very low, with a value down to approximately 10^−13^ A over the entire *V*_CE_ range. This low *I*_Cdark_ confirms the defect-free crystal quality after the wafer bonding and ELO processes. In contrast to the 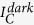 characteristics, the 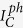 characteristics were quite different between the two devices. At a high bias condition of *V*_CE_ > 0.25 V, 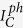 of the HPT fabricated using the ultra-fast ELO process was almost twice as high as that of the HPT fabricated using the conventional ELO process. This difference is attributed to the small amount of etching residues for the ultra-fast ELO process, which enables a lower contact resistance of the collector contact. Figure 5e shows the optical gain (*G*_opt_ = hυΔ*I*_c_/q*P*_in_, where hυ and Δ*I*_c_ are the incident photon energy and the difference between 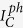 and 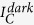, respectively) of the two devices. As estimated from the 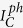 characteristics, the *G*_opt_ of the HPT fabricated using the ultra-fast ELO process shows higher values, demonstrating another benefit of the developed ELO process besides the time reduction. Furthermore, array formation with each HPTs using the developed process can be expanded to the focal plane array for the high resolution^40^ and wide angle detectors^41^.

In conclusion, we have demonstrated a new integration concept of a high-throughput fabrication process for III-V/Si structures with a high-quality film through use of the wafer bonding and ultra-fast ELO processes. Donor wafer re-usability was also confirmed after the ELO process. These results promise dramatic cost reduction in the production of high-quality III-V on Si structures, which expand the use of the III-V and extend the conventional Si-based semiconductor industry. Further combination with a multiple epitaxial transfer^42^ should be a strong component of technologies in the future III-V/Si device era.

## Method

### Epitaxial growth

A 2-inch GaAs wafer (001) was prepared for an epitaxial growth for all experiments. The epitaxial layers of the GaAs HEMTs and InGaP/GaAs HPTs were grown by solid source molecular beam epitaxy (MBE) with the use of Si and Be cell as sources of n- and p-type dopants. GaAs HEMTs were composed of an n-type contact layer, an Al_0.3_Ga_0.7_As barrier layer, and a GaAs channel layer. InGaP/GaAs HPTs were composed of an n-type GaAs collector contact layer, an n-type InGaP etch stop layer, an n-type GaAs collector, a p-type GaAs base, an n-type InGaP emitter, an n-type InGaP subemitter, an n-type GaAs emitter contact layer, and an n-type InGaAs capping layer. The epitaxial layers of GaAs solar cells were grown by metal-organic chemical vapour deposition (MOCVD) with AsH_3_, PH_3_, TMGa, TMIn, and TMAl as the precursors. SiH_4_ and DMZn were used for n- and p-type dopants. The GaAs solar cells were composed of a p-type GaAs contact layer, and a p-type InGaP back surface field layer, a p-type GaAs base, an n-type GaAs emitter, an n-type GaAs window layer, and n-type GaAs contact layer. For the ELO process, an Al_0.85_Ga_0.15_As sacrificial layer was inserted between the device layer and the GaAs donor wafer. Optionally, an etch stop layer of InGaP/GaAs was also inserted between the Al_0.85_Ga_0.15_As layer and the device layer and also between the Al_0.85_Ga_0.15_As layer and the GaAs donor wafer. To check the quality of the epitaxial film, Hall measurement was carried out with 0.7 × 0.7 cm^2^ pieces at 0.55 T magnetic field using HMS 3000 system, Ecopia. During the measurement, no bias gating was applied.

### Wafer bonding and epitaxial lift off

III-V and Si wafers were cleaned by NH_4_OH and HF solutions, respectively, to remove the native oxides. Next, bonding materials, i.e., Y_2_O_3_ or Pt/Au in this study, were deposited via electron beam evaporation. A pre-patterning process was performed using H_3_PO_4_- and HCl-based solutions for the etching of Al(Ga)As and InGaP, respectively. Subsequently, the wafers were cleaned by acetone in an ultra-sonic bath, followed by surface plasma treatment using O_2_ and Ar for Y_2_O_3_ and Pt/Au, respectively. The surface-treated wafers were bonded to each other in the air, followed by uniaxial pressing with a force of 180 kgf. The ELO process was performed in HF-based solutions until the separation of the III-V/Si and the GaAs donor wafer was completed. The completion of ELO was determined when III-V/Si wafer and III-V donor wafer were separated. Here, we observed the sample separation on every 1 min after dipping the samples in HF-based solutions.

### GaAs HEMTs

Mesa etching was performed using a H_3_PO_4_-based solution, followed by Y_2_O_3_ field-oxide deposition. S/D contacts were formed by Pd/Ge/Au deposition and rapid thermal annealing (RTA) at 150 °C for 3 hours. Next, the contact layer was selectively etched by citric acid:H_2_O_2_ (3:1 volume ratio) solutions for the gate electrode formation. Finally, the gate electrode of Ti/Pt/Au was deposited. Each device was characterized using a semiconductor parameter analyser (Hewlett Packard, 4156 A) in the dark.

### GaAs solar cells

After the wafer bonding with a Pt/Au bonding material, the Pt/Au layer acted as a bottom electrode for the GaAs solar cells. The top electrode of Ni/Au/Ge/Ni/Au was deposited, followed by RTA at 400° C for 40 sec. Next, the n-type GaAs contact layer was selectively etched using H_3_PO_4_-based solutions through the top electrode as a mask. Each device was characterized using a solar simulator (McScience, XES-301 S), IPCE (McScience, K3100 Spectral IPCE Measurement system).

### InGaP/GaAs HPTs

Similar to the GaAs solar cells, the Pt/Au layer acted as an emitter electrode (located at the bottom) after the ELO process. The top electrode of Ni/Au/Ge/Ni/Au was deposited, followed by RTA at 400 °C for 40 sec. Next, the n-type GaAs collector contact layer was selectively etched using H_3_PO_4_-based solutions through the top electrode as a mask. The optical performances of the fabricated devices were characterized utilizing a 635-nm laser diode and a semiconductor parameter analyser (Hewlett Packard, 4156 A). For laser power calibration, the incident optical power through a lensed fiber was measured using an optical meter (Newport, 1835-C) equipped with a silicon photodetector (Newport, 818-UV/DB) in the dark.

## Additional Information

**How to cite this article**: Geum, D.-M. *et al.* Ultra-high-throughput Production of III-V/Si Wafer for Electronic and Photonic Applications. *Sci. Rep.* **6**, 20610; doi: 10.1038/srep20610 (2016).

## Supplementary Material

Supplementary Information

## Figures and Tables

**Figure 1:**
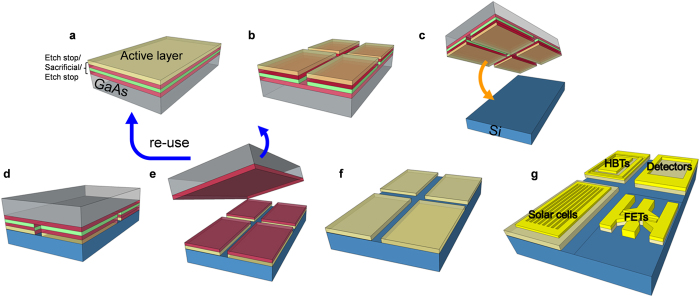
Schematic fabrication process of GaAs/Si structure. (**a**) Epitaxial growth of III−V layers. (**b**) Bonding material deposition, followed by the pre-patterning step. (**c**) Surface treatment and wafer bonding. (**d**) Bonded III-V/Si wafer. (**e**) ELO in HF-based solutions; here, the separated donor wafer is re-used. (**f**) III-V/Si wafer for the device fabrication. (**g**) Device fabrication, e.g., HEMTs, solar cells, HPTs, etc.

**Figure 2:**
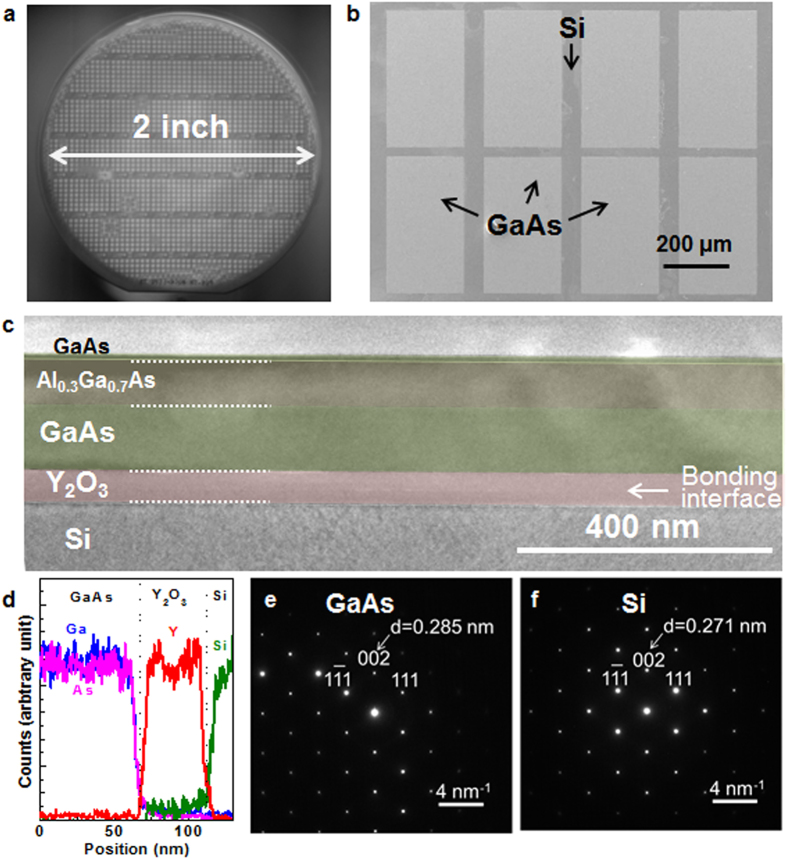
GaAs-OI on a Si substrate via the wafer bonding and ELO processes. (**a**) IR image of a GaAs-OI on Si sample after the wafer bonding. Patterned GaAs arrays were securely bonded onto the Si substrate. (**b**) Typical SEM image of a GaAs-OI on Si sample. GaAs pattern arrays are formed on the Si substrate. (**c**) Cross-sectional TEM image of a GaAs-OI on Si sample, showing uniform GaAs HEMT layers on Si. The GaAs HEMT is composed of a GaAs contact layer, an Al_0.3_Ga_0.7_As barrier layer, and a GaAs channel layer. (**d**) EDAX profiles for Ga (blue), As (pink), Y (red), and Si (Green) evaluated along the fabricated substrate. (**e)** TED pattern of GaAs region. (**f**) TED pattern of Si region.

**Figure 3:**
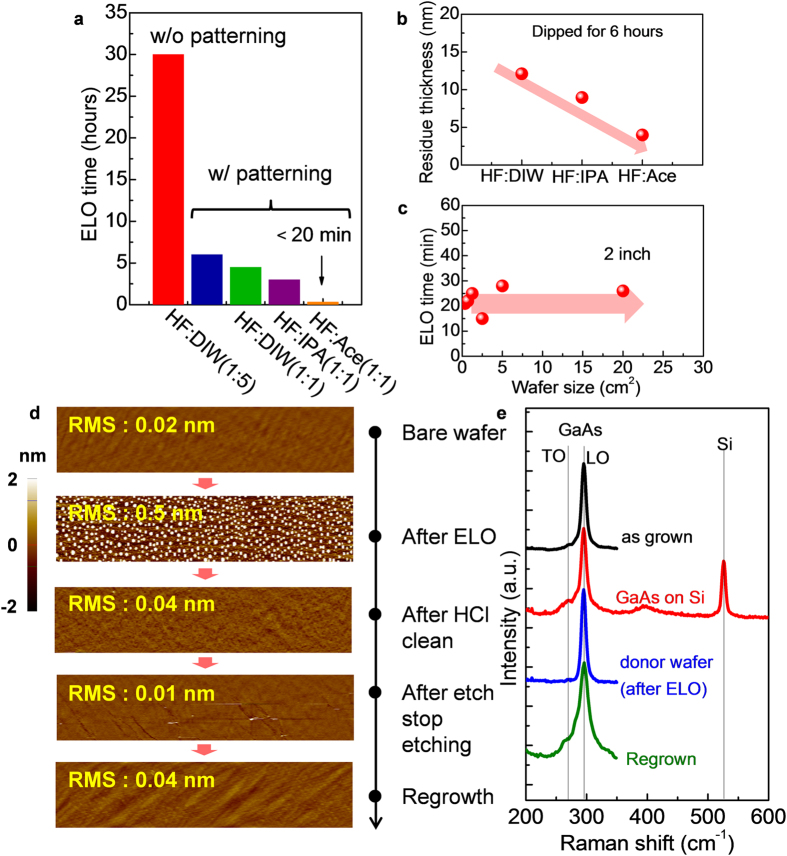
Ultra-fast ELO and wafer re-use after the ELO. (**a**) Etching solution dependence of the ELO time as a parameter of the pre-patterning process. With the pre-patterning and the insertion of the etching acceleration solutions, the ELO time was significantly reduced. (**b**) Etching solution dependence of the residue thickness. (**c**) The effect of a wafer size for the ELO process; a negligible dependence was observed. (**d)** AFM images of GaAs surface at each process step. (**e)** Raman spectra of the GaAs layer at each process step.

**Figure 4:**
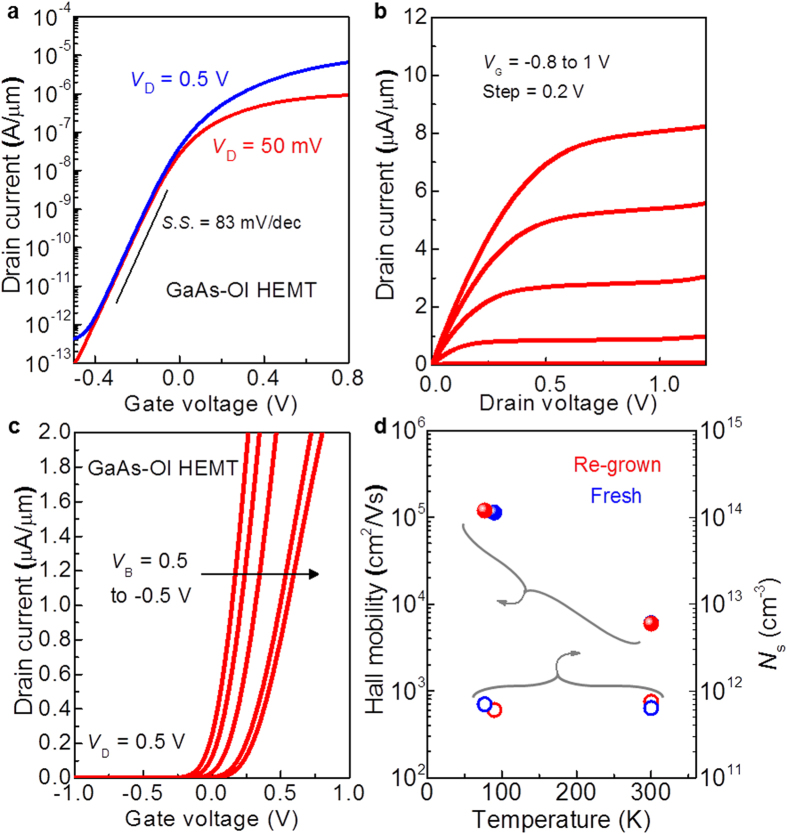
Performance of the GaAs-OI HEMTs and wafer re-usability after the ELO process. (**a)** Measured transfer characteristics of a GaAs-OI HEMT with a *L*_G_ of 2 μm. The device exhibits a quite low S.S. of 83 mV per decade, which is close to the theoretical limit of 60 mV per decade at room temperature. (**b)** Output characteristics of the same device shown in (**a**) showing clear current saturation. (**c)** *I*_D_-*V*_G_ curves for different *V*_B_; the electrical characteristics of the GaAs-OI HEMT can be controlled by *V*_B_ after the device fabrication. (**d)** Temperature dependence of the Hall mobility and *N*_s_ of the fresh and re-grown GaAs HEMTs.

**Figure 5:**
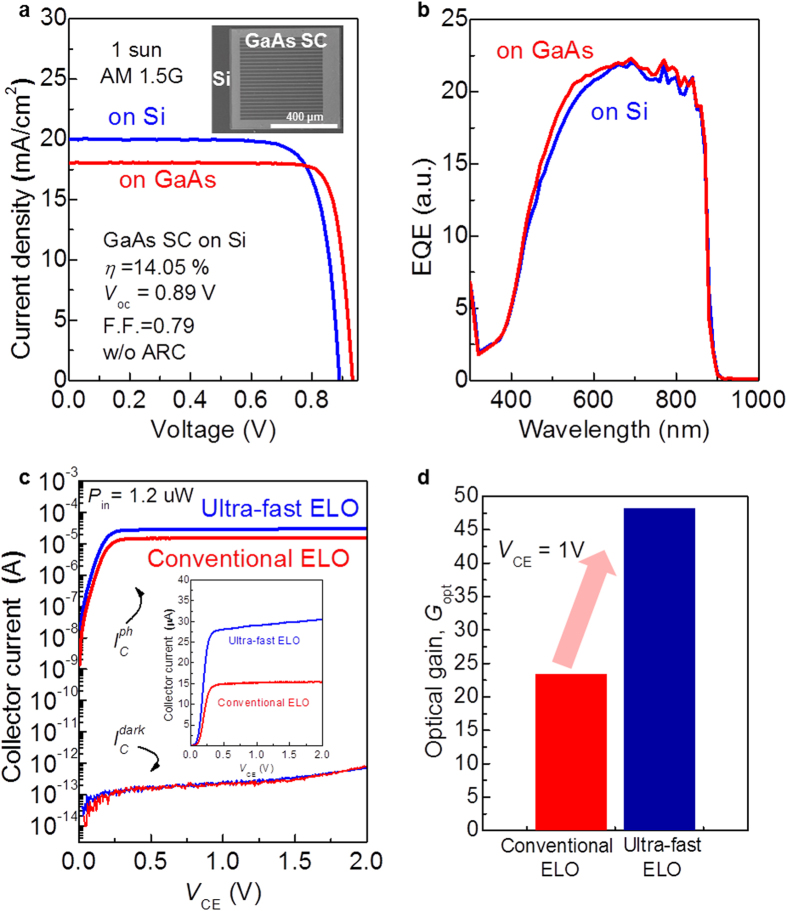
GaAs solar cells and InGaP/GaAs HPTs on Si. (**a**) *J*-*V* characteristics of the GaAs solar cell on Si (blue) and GaAs (red, control) under AM 1.5 G, 1-sun measurement condition. The inset is a top view SEM image showing the fabricated GaAs solar cell on Si. (**b)** EQE spectra of the GaAs solar cell on Si (blue) and GaAs (red). (**c)** *I*_Cdark_ and *I*_Cph_ characteristics of the InGaP/GaAs HPT on Si as a function of *V*_CE_. (**d)** Incident optical power dependence of the *G*_opt_ of the same device shown in (**c)**.
