# Supplementary material for: Ultra-high-throughput Production of III-V/Si Wafer for Electronic and Photonic Applications
Source: Sci Rep. 2016 Feb 11;6:20610. doi: 10.1038/srep20610 (PMC4750074; doi:10.1038/srep20610)
Supplement: Supplementary Information [file srep20610-s1.doc]

**Supplementary Information**

**Ultra-high-throughput production of III-V/Si wafer for III-V electronic and photonic applications**

Dae-Myeong Geum^1, 2^, Min-Su Park^1^, Ju Young Lim^3^, Hyun-Duk Yang^1^, Jin Dong Song^1^, Chang Zoo Kim^4^, Euijoon Yoon^2^, SangHyeon Kim^1^*, and Won Jun Choi^1^

^1^ Korea Institute of Science and Technology (KIST), Hwarangno 14-gil 5, Seongbuk-gu, Seoul, 136-791, Korea

^2^ Seoul National University, Gwanak-gu, Seoul, 151–742, Korea

^3^ Korea Photonics Technology Institute (KOPTI), Cheomdan venture-ro 108-gil 9, Buk-gu, Gwanju-si, 500-799, Korea

^4^ Korea Advanced Nanofab Center (KANC), Gwanggyo-ro 109, Yeongtong-gu, Suwon-si, Gyeonggi-do, 443-270, Korea

**E-mail: sh-kim@kist.re.kr*

**Contents**

- Estimation of the production cost of III-V/Si wafer
- Thermal expansion issue for III-V/Si
- TEM images of GaAs HEMT on Si
- Pattern dimension effects on the ELO time
- Epitaxial growth of the III-V layer
- Wafer bonding and epitaxial lift-off
- Surface morphology of Y_2_O_3_/Si and Au/Pt/Si
- Fabrication of GaAs HEMTs
- Fabrication of GaAs solar cells
- SEM images of the samples during the solar cell fabrication
- Fabrication of GaAs HPTs

**Estimation of the production cost of III-V/Si wafer**

To examine the cost efficiency of our transfer approach, we calculated the expected cost reduction comparing to conventional wafer bonding/donor wafer etching and selective growth (aspect ratio trapping (ART)) approach.

First, we assumed that the total cost (C_t_) is composed of the wafer cost (C_wafer_), the system cost (C_system_), and the material cost (C_material_).

C_t_ = C_wafer_ + C_system_ + C_material_

To estimate the cost and throughput of III-V/Si wafer using our wafer bonding and ELO techniques, we defined parameters as follows :

T_lo_ : Time to load and unload a substrate in the growth chamber (min)

T_act_ : Time to grow an active layer (min)

T_sac_ : Time to grow a sacrificial layer (min)

T_buffer_ : Time to grow a buffer layer (min)

T_etch_ : Time to etch all donor substrate (min)

T_elo_ : Time to complete ELO process (min)

R_op_ : Running operation cost per time ($/h)

C_act_ : Cost of source materials to grow an active layer ($)

C_sac_ : Cost of source materials to grow a sacrificial layer ($)

C_buffer_ : Cost of source materials to grow a sacrificial layer ($)

C_donor_ : Cost of a GaAs donor wafer ($)

C_sil_ : Cost of a Si substrate for layer transfer ($)

C_re-use_ : Cost of processing to re-use the donor wafer ($)

N : Number of the production wafer

Here, we consider three kinds of integration method of III-V/Si, which are our approach of wafer bonding/ELO, the wafer bonding/donor wafer etching approach, and selective epitaxy approach (ART).

To estimate the C_t_ to produce the same amount of wafers, we calculated the processing time of each approaches.

T_our_ (Time for our approach of wafer bonding /ELO) = T_lo_ + T_act_ + T_sac_ + T_elo_

T_con_ (Time for conventional approach of wafer bonding/etching) = T_lo_ + T_act_ + T_sac_ + T_etch_

T_ARC_ (Time for ART approach) = T_lo_ + T_act_ + T_lo_ + T_buffer_

Here, T_ARC_ include the double of T_lo_. It is because that ARC approach needs chemical mechanical polishing between the growth of buffer layer and the growth of active layer (N. Waldron *et al.,* *ECS Transactions* **45,** p. 115-128 (2012)). Therefore, we assumed T_ARC_ need the double of T_lo_.

Then, C_system_, C_wafer_, and C_material_ can be expressed by following table.

|  | Our approach | Conventional wafer bonding/donor wafer etching | ART |
| --- | --- | --- | --- |
| C_system_ | T_our_ × R_op_ | T_con_ × R_op_ | T_ARC_ × R_op_ |
| C_wafer_ | C_donor_ + (C_re-use_ + C_sil_) × N | (C_donor_ + C_sil_) × N | C_sil_ × N |
| C_material_ | (C_act_ + C_sac_) × N | (C_act_ + C_sac_) × N | (C_act_ + C_buffer_) × N |

As the key parameters, we defined the time and cost as follows from [Ref. J. Yoon *et al.*, Nature **465**, p. 329 (2010), EPIWORKS].

T_lo_ = 60 min

T_act_ = 1.67 min (thickness = 100 nm, growth rate ~ 1 nm/sec)

T_sac_ = 0.17 min (thickness = 10 nm)

T_buffer_ = 2.5 min (thickness = 150 nm)

T_etch_ = 120 min (Etch rate = 5 μm/min, wafer thickness = 700 μm)

T_elo_ = 20 min

R_op_ = 125 $/hour

C_act_ = 0.21 $ (GaAs 100 nm, 2.11 $/μm)

C_sac_ = 0.022 $ (AlGaAs 10 nm, 2.20 $/μm)

C_buffer_ = 0.3165 $ (150 nm, 2.11 $/μm)

C_donor_ = 150 $ (6 inch GaAs)

C_sil_ = 10 $ (6 inch Si)

C_re-use_ = 10 $

With the parameters described above, we calculated the production cost using each integration approaches as shown in following figure. Total production cost is shown in the left figure and the production cost per one wafer is shown in the right figure as a function of N. Even though cost benefit is not so high with small N, the cost benefit of our approach (wafer bonding and ELO) increases with increasing N. Even just for one wafer production, our approach is still economical comparing to the conventional wafer bonding/donor wafer etching approach due to the time reduction via developed ELO process. With increasing N, cost reduction is more efficient by reducing wafer cost via the re-use of the donor wafer. Even comparing to ART approach, our approach will be more economical at high N due to the processing time reduction and the cut of the wafer cost. At large N, cost reduction factor reaches 0.35 and 0.71 comparing to conventional wafer bonding/donor wafer etching approach and ART approach, respectively.


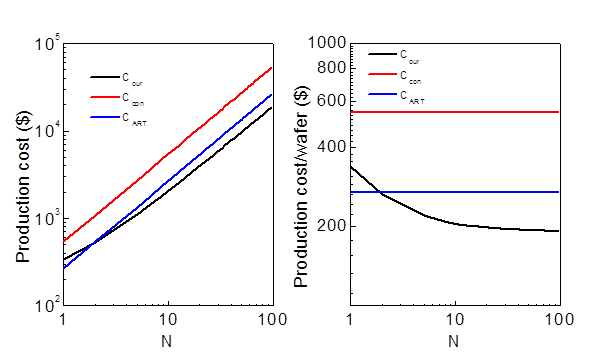


**Figure S1. Estimation of the production cost III-V/Si wafer.** **a,** Production cost as a function of N. **b,** Production cost per one wafer as a function of N.

Not only the cost reduction, there is one more significant benefit using our approach. Reciprocal value of T_our_, T_con_, T_ARC_ correspond to the throughput of each approach. Using our approach, 2.25 and 1.52 times higher production throughput can be achieved comparing to the conventional wafer bonding/donor wafer etching approach and ART approach, respectively.

Following table highlighted the enhancement factor of our approach. Through the cost reduction and throughput improvement, our approach provides the enhancement factor of about 7 × and 2 × comparing to the conventional wafer bonding/donor wafer etching approach and ART approach, respectively.

In our approach, further improvement is expected combining multilayer bonding technique shown from the Prof. Rogers group (J. Yoon *et al.*, Nature **465**, p. 329 (2010)) and multi wafer growth techniques using mass-production CVD.

|  | Our approach | Conventional wafer bonding/donor wafer etching | ART |
| --- | --- | --- | --- |
| Cost factor (A) | 1 | 2.83 | 1.40 |
| Throughput increase factor (B) | 1 | 0.44 | 0.67 |
| Total enhancement factor (B/A) | 1 | 0.15 | 0.48 |

**Thermal expansion issue for III-V/Si**

For the device fabrication using GaAs on Si structure, one important thing should be considered is the difference of the thermal expansion coefficient between GaAs and Si. We roughly estimated an acceptable thickness of GaAs layer on Si substrate considering the strain induced by a thermal expansion. Here, we did not consider the effects of the pattern size/shape and bonding intermediate. An acceptable thickness of GaAs layer on Si substrate is quite thin. Therefore, a processing temperature including wafer bonding and device fabrication should be minimized.

**Figure S2. Calculated critical thickness of GaAs layer on Si as a function of temperature considering induced strain by the difference of thermal expansion**

T**EM images of GaAs HEMT on Si**

To clearly show the TEM image, we provide an unprocessed TEM of bonded sample of GaAs HEMT on Si. Each was clearly identified in the TEM image.


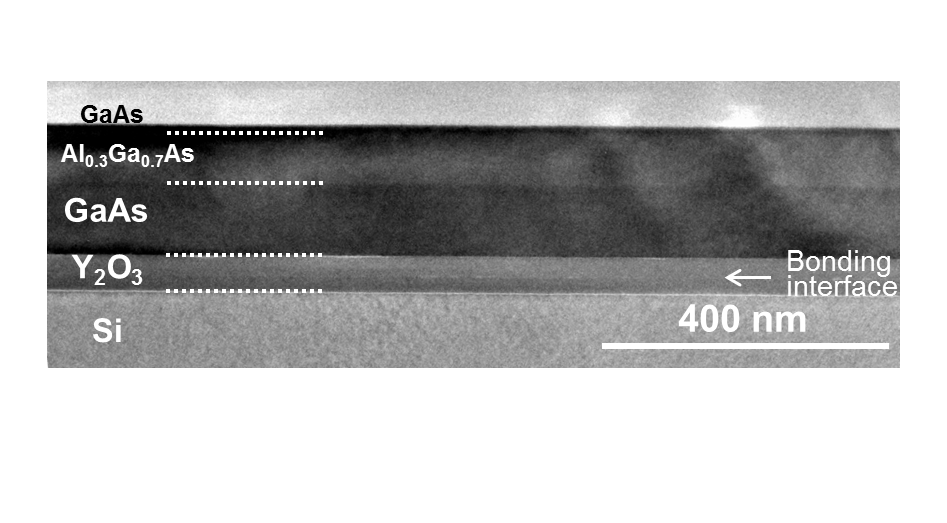


**Figure S3. Cross-sectional TEM image of a GaAs-OI on Si.** The GaAs HEMT is composed of a GaAs contact layer, an Al_0.3_Ga_0.7_As barrier layer, and a GaAs channel layer.

**Pattern dimension effects on ELO time**

Pattern dimension is quite important parameter for our ELO with pre-patterning. Therefore, we investigated dimension dependence of ELO time.


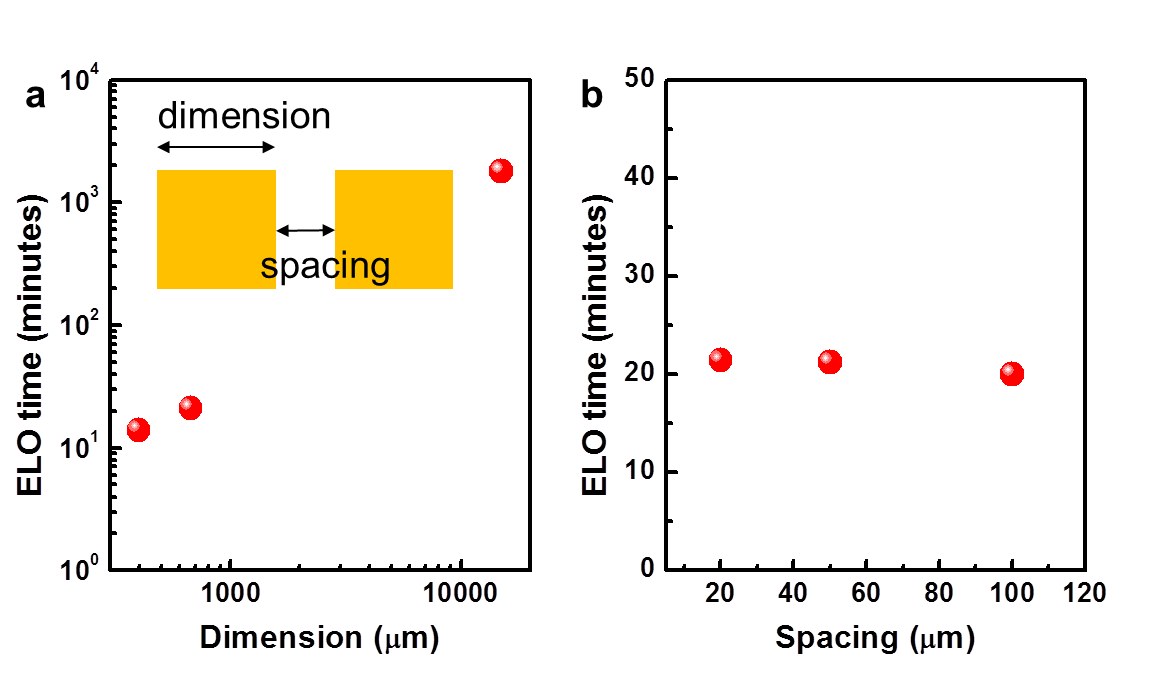


**Figure S4. Dimension dependence of ELO time.** **a,** Mesa size dependence of ELO time. Significant time reduction was obtained with a decrease of the size. **b,** Spacing dependence of ELO time. There was almost no spacing dependence.

**Epitaxial growth of the III-V layer**

The epitaxial III-V layers were grown on 2-inch GaAs wafer (001) for all experiments. The epitaxial layers of the GaAs HEMTs and InGaP/GaAs HPTs were grown by solid source molecular beam epitaxy (MBE) with the use of Si and Be cell as sources of n- and p-type dopants. The epitaxial layers of the GaAs solar cells were grown by metal-organic chemical vapor deposition (MOCVD) with AsH_3_, PH_3_, TMGa, TMIn, and TMAl as the precursors. SiH_4_ and DMZn were used for n- and p-type dopants. The details of epitaxial structures are shown as follows.


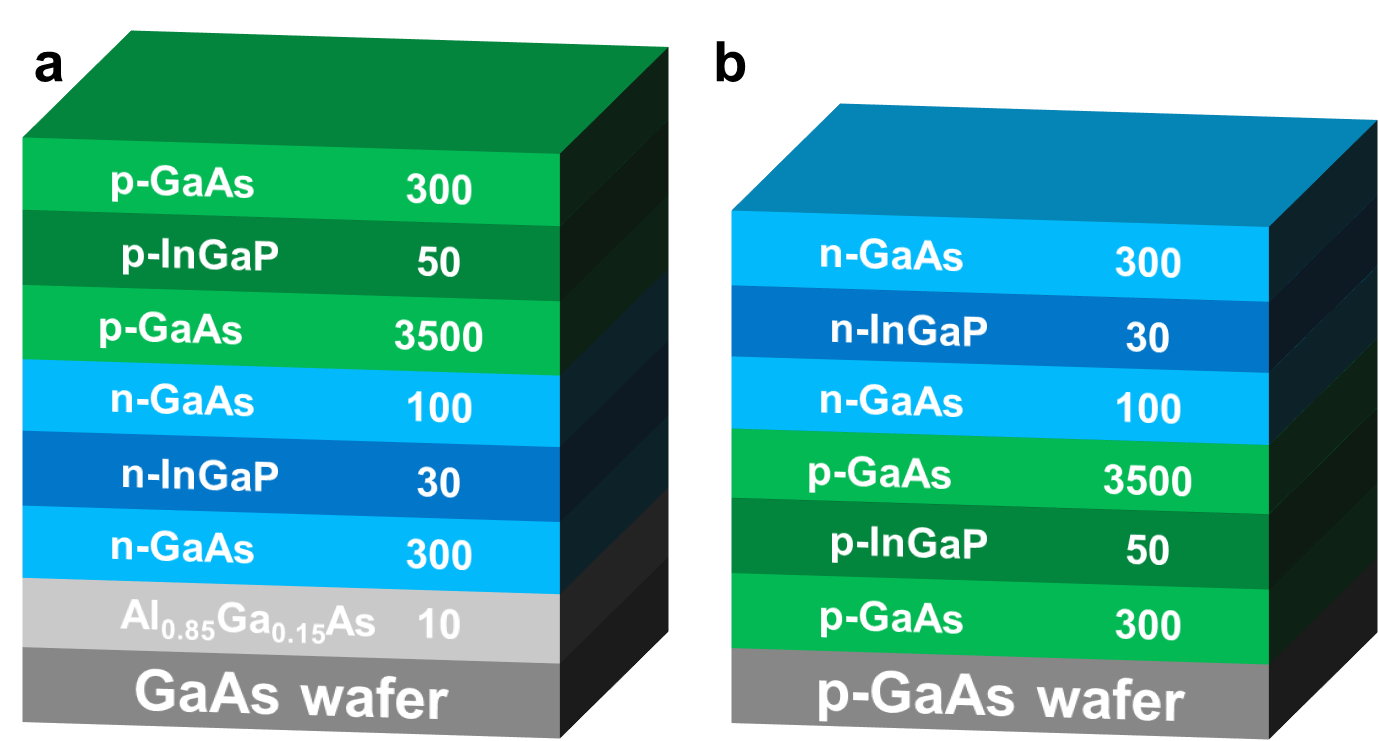


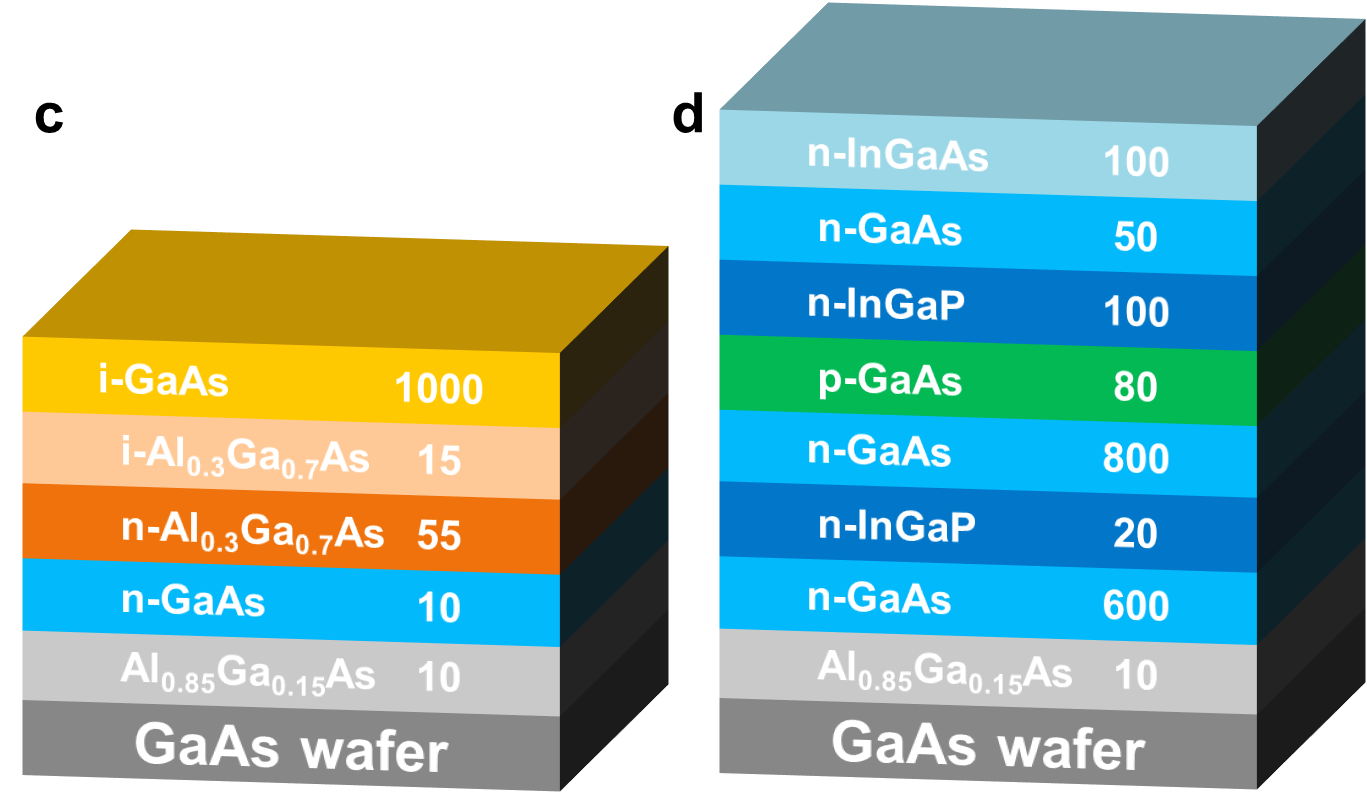


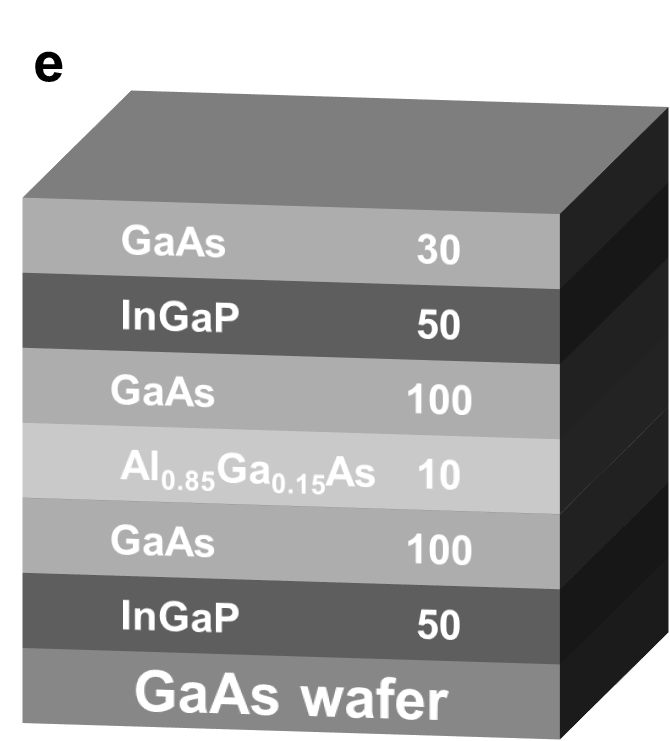


**Figure S5. The epitaxial structures of the III-V layers used in this study.** **a,** Schematic epitaxial structure of the inverted GaAs solar cell for the ELO. **b,** Schematic epitaxial structure of the GaAs solar cell on GaAs (control). **c,** Schematic epitaxial structure of the inverted GaAs HEMT for the ELO. **d,** Schematic epitaxial structure of the inverted the InGaP/GaAs HPT for the ELO. **e,** Schematic epitaxial structure for the ELO with a double etch stop layers. The layer thickness is shown in the right side in each layer with nm scale.

**Wafer bonding and ELO**

The wafer bonding and ELO processes were carried out following the process flow shown in Fig. S2. Here, two types of a bonding material are shown. Figure S2a, b show the case of Y_2_O_3_ and Pt/Au as a bonding material, respectively.


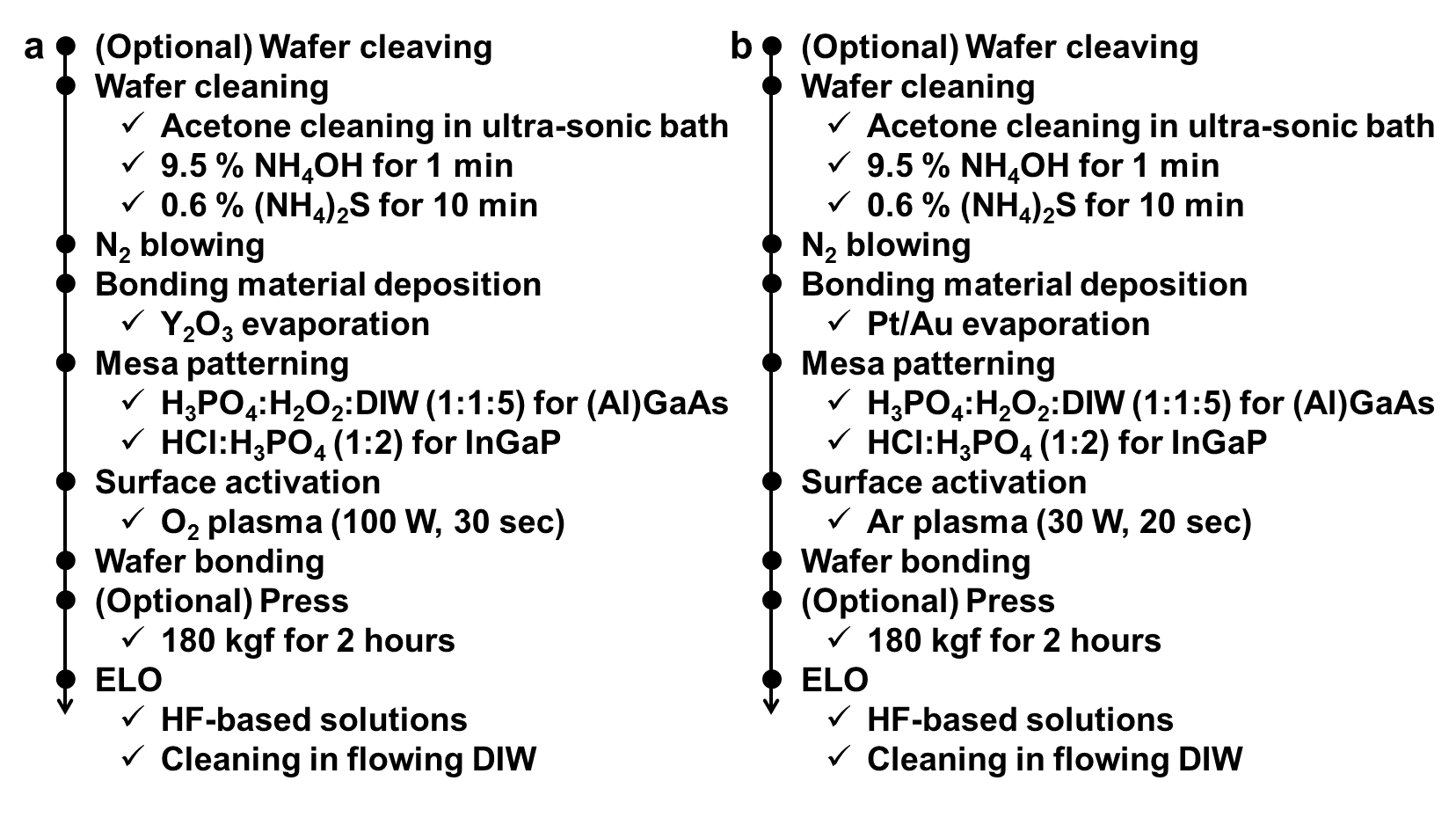


**Figure S6. Process flow of the wafer bonding and ELO process.** **a,** Process details of the wafer bonding and ELO with Y_2_O_3_ as a bonding material. **b,** Process details of the wafer bonding and ELO with Pt/Au as a bonding material

**Surface morphology of** **Y_2_O_3_/Si and Au/Pt/Si**

The surface of Y_2_O_3_/Si and Au/Pt/Si were measured by AFM. AFM images are shown in Fig. S3. RMS roughness of all samples was quite small of < 0.1 nm, which is ultra-flat and smooth enough for the wafer bonding.


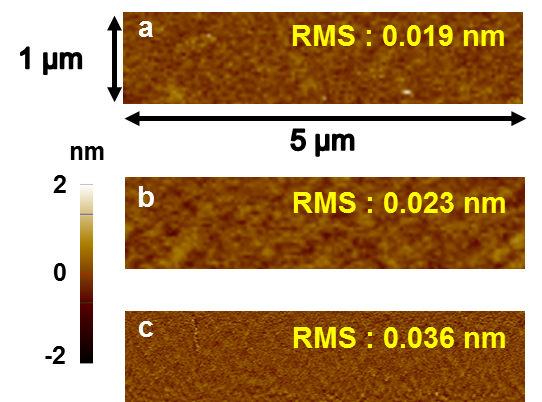


**Figure S7. Surface morphology of Y_2_O_3_/Si and Au/Pt/Si.** **a,** AFM image of the 2-nm-thick Y_2_O_3_/Si. **b,** AFM image of the 10-nm-thick Y_2_O_3_/Si. **c,** AFM image of the 10-nm-thick Au/10-nm-thick Pt/Si.

**Fabrication of GaAs HEMTs**

The GaAs HEMTs were fabricated with the process shown in Fig. S4. Figure S4 also shows the final schematic device structure. Through the back-gate of Si, device characteristics can be controlled after the device fabrication.


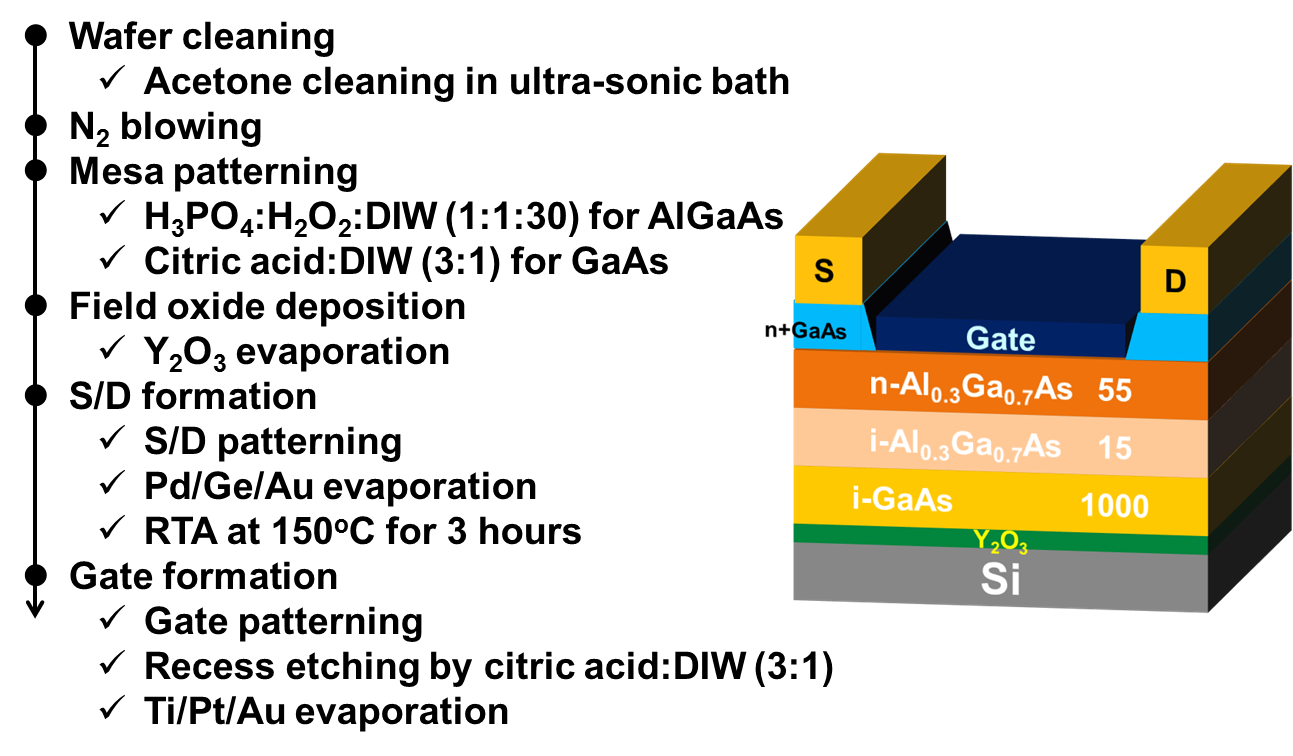


**Figure S8. GaAs HEMT on Si.** Process details for the fabrication of the GaAs HEMTs on Si.

**Fabrication of GaAs solar cells**

The GaAs solar cells were fabricated with the process shown in Fig. S5. Figure S5 also shows the final schematic device structure. Here, device isolation process by the mesa etching could be skipped due to the use of the ELO with a pre-patterning.


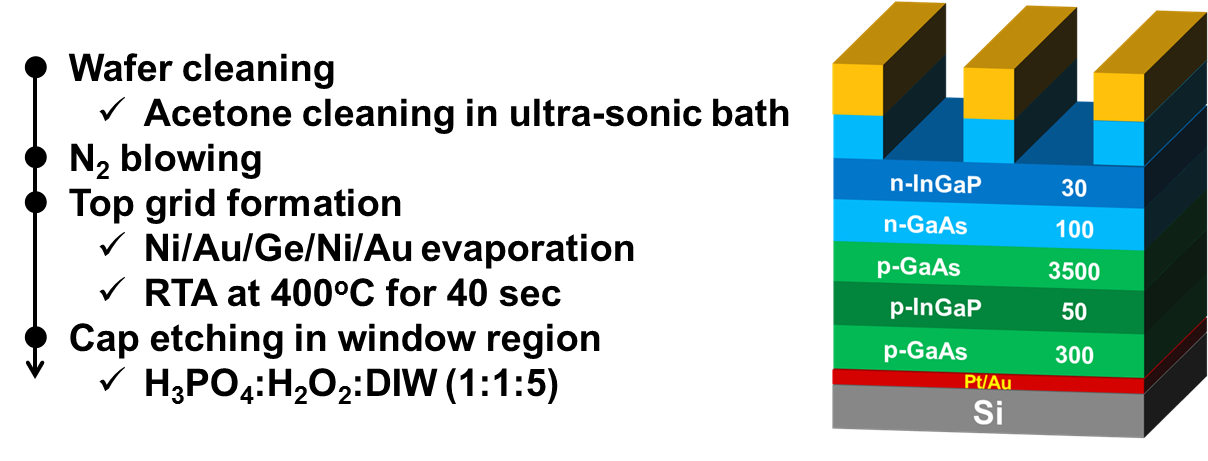


**Figure S9. GaAs solar cell on Si.** Process details for the fabrication of the GaAs solar cells on Si

**SEM images of the samples during the solar cell fabrication**

During the solar cell fabrication, SEM images were measured at each process step. SEM images are shown in Fig. S6. Cell arrays are clearly formed on the Si as shown in Fig. S6a, and b through the wafer bonding and ELO processes. SEM images of the GaAs solar cell on Si are shown in Fig. S6c and d after the fabrication.


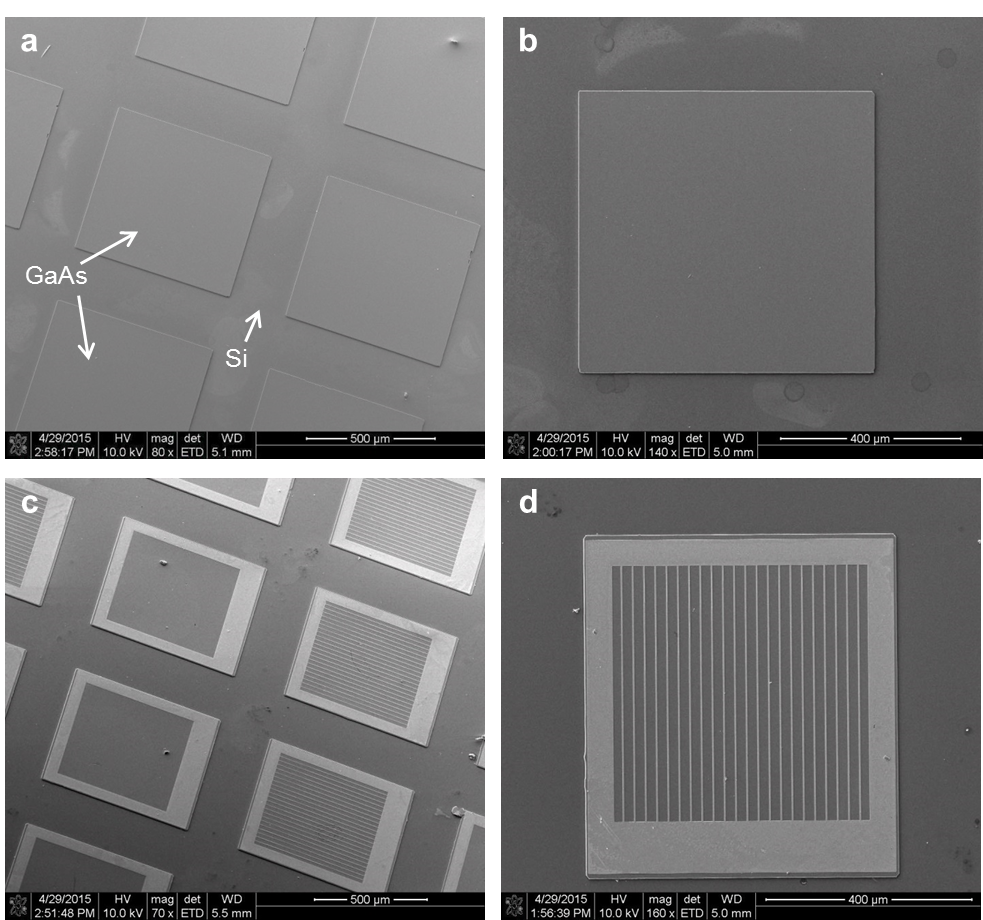


**Figure S10. GaAs solar cells on Si.** **a,** SEM image of the epitaxial layer of the GaAs solar cell on Si just after the wafer bonding and ELO. **b,** SEM image of one cell of the same sample shown in **a**. **c,** SEM image of the GaAs solar cell on Si after complete device fabrication. **d,** SEM image of one cell of the same device shown in **c**.

**Fabrication of InGaP/GaAs HPTs**

The InGaP/GaAs HPTs were fabricated with the process shown in Fig. S7. Figure S7 also shows the final schematic device structure. Metal of Pt/Au used as a bonding material also roles as a back mirror for the photon recycling.


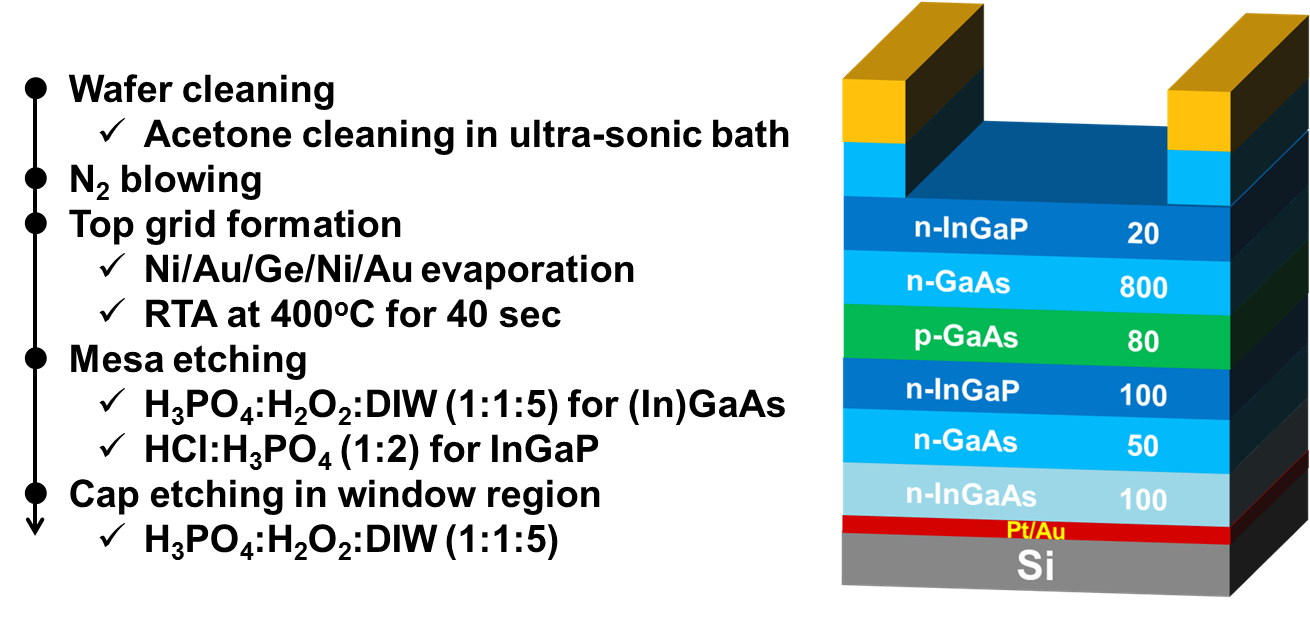


**Figure S11. InGaP/GaAs HPT on Si.** Process details for the fabrication of the InGaP/GaAs HPT on Si
